# Fine-Scale Genetic Structure and Natural Selection Signatures of Southwestern Hans Inferred From Patterns of Genome-Wide Allele, Haplotype, and Haplogroup Lineages

**DOI:** 10.3389/fgene.2021.727821

**Published:** 2021-08-24

**Authors:** Mengge Wang, Didi Yuan, Xing Zou, Zheng Wang, Hui-Yuan Yeh, Jing Liu, Lan-Hai Wei, Chuan-Chao Wang, Bofeng Zhu, Chao Liu, Guanglin He

**Affiliations:** ^1^Guangzhou Forensic Science Institute, Guangzhou, China; ^2^Faculty of Forensic Medicine, Zhongshan School of Medicine, Sun Yat-sen University, Guangzhou, China; ^3^Department of Forensic Medicine, College of Basic Medicine, Chongqing Medical University, Chongqing, China; ^4^College of Basic Medicine, Chongqing University, Chongqing, China; ^5^Institute of Forensic Medicine, West China School of Basic Science and Forensic Medicine, Sichuan University, Chengdu, China; ^6^School of Humanities, Nanyang Technological University, Singapore, Singapore; ^7^State Key Laboratory of Marine Environmental Science, State Key Laboratory of Cellular Stress Biology, Department of Anthropology and Ethnology, Institute of Anthropology, National Institute for Data Science in Health and Medicine, School of Life Sciences, Xiamen University, Xiamen, China; ^8^Department of Forensic Genetics, School of Forensic Medicine, Southern Medical University, Guangzhou, China; ^9^Key Laboratory of Shaanxi Province for Craniofacial Precision Medicine Research, College of Stomatology, Xi’an Jiaotong University, Xi’an, China; ^10^Clinical Research Center of Shaanxi Province for Dental and Maxillofacial Diseases, College of Stomatology, Xi’an Jiaotong University, Xi’an, China

**Keywords:** allele-sharing, admixture history, Han Chinese, haplotype chunk, genetic origin, nature selection

## Abstract

The evolutionary and admixture history of Han Chinese have been widely discussed *via* traditional autosomal and uniparental genetic markers [e.g., short tandem repeats, low-density single nucleotide polymorphisms). However, their fine-scale genetic landscapes (admixture scenarios and natural selection signatures) based on the high-density allele/haplotype sharing patterns have not been deeply characterized. Here, we collected and generated genome-wide data of 50 Han Chinese individuals from four populations in Guizhou Province, one of the most ethnolinguistically diverse regions, and merged it with over 3,000 publicly available modern and ancient Eurasians to describe the genetic origin and population admixture history of Guizhou Hans and their neighbors. PCA and ADMIXTURE results showed that the studied four populations were homogeneous and grouped closely to central East Asians. Genetic homogeneity within Guizhou populations was further confirmed *via* the observed strong genetic affinity with inland Hmong-Mien people through the observed genetic clade in Fst and outgroup *f*_3_*/f*_4_-statistics. qpGraph-based phylogenies and *f*_4_-based demographic models illuminated that Guizhou Hans were well fitted *via* the admixture of ancient Yellow River Millet farmers related to Lajia people and southern Yangtze River farmers related to Hanben people. Further ChromoPainter-based chromosome painting profiles and GLOBETROTTER-based admixture signatures confirmed the two best source matches for southwestern Hans, respectively, from northern Shaanxi Hans and southern indigenes with variable mixture proportions in the historical period. Further three-way admixture models revealed larger genetic contributions from coastal southern East Asians into Guizhou Hans compared with the proposed inland ancient source from mainland Southeast Asia. We also identified candidate loci (e.g., MTUS2, NOTCH4, EDAR, ADH1B, and ABCG2) with strong natural selection signatures in Guizhou Hans *via* iHS, nSL, and ihh, which were associated with the susceptibility of the multiple complex diseases, morphology formation, alcohol and lipid metabolism. Generally, we provided a case and ideal strategy to reconstruct the detailed demographic evolutionary history of Guizhou Hans, which provided new insights into the fine-scale genomic formation of one ethnolinguistically specific targeted population from the comprehensive perspectives of the shared unlinked alleles, linked haplotypes, and paternal and maternal lineages.

## Introduction

Southwestern East Asia is one of the most ethnolinguistically diverse regions around the world. Genetic origin, subsequent migration, isolation, plausible admixture, and local adaptation history of ethnolinguistic southern Chinese populations were widely discussed *via* different genetic markers, mainly including autosomal short tandem repeats (STRs), single nucleotide polymorphism (SNPs), and copy number variations (CNVs) (Chen et al., 2019; Zhang C. et al., 2019; He et al., 2021; Liu et al., 2021a). However, most of these studies focused on the genetic variations and forensic features of low-density genetic markers in the Han Chinese populations. Genome-wide data of Han people were relatively inefficient considering their largest population size and widely geographically distributed features. Previous chip-based population genetic analysis from the southernmost Han Chinese in Hainan Province revealed that these Han Chinese harbored more genetic materials from surrounding indigenous people (Austronesian, Austroasiatic, Tai-Kadai, and Hmong-Mien speakers) (He G. et al., 2020). Additionally, genetic admixture history from northern Hans and northwestern Hans also revealed that extensive admixture events, including the ancestral sources related to the southern East Asians, southern Siberians, and limited but important ancestral sources linked to the western Eurasians, participated in their genomic formation processes (He G.-L. et al., 2020; Yao et al., 2021). There are also important studies focused on the genetic relationships between central Hans and their neighbors (e.g., Han, Manchu, Mongolian, and Tujia). However, all of these studies mainly focused on the patterns from the shared alleles and sample frequency spectrum of independent SNPs (Chen et al., 2021; He et al., 2021) and lacked evidence from a fine-scale genetic structure based on the shared haplotype chunks (successive linked SNP fragments) and uniparental haplogroup lineages.

A previous genetic study based on the frequency spectrum of maternal and paternal founding lineages from the Neolithic to historical populations in North China has found that extensive population movement and admixture occurred here (Chen et al., 2019). Recently, admixture history and genetic structure patterns of modern and ancient East Asians were also discussed and characterized *via* genome-wide ancient DNA data. These earlier findings extracted from ancient genomes documented that the gene flow from the behaviorally and anatomically modern human flowed into Southeast Asians over 65 thousand years ago (kya), which had left major genetic traces in modern Austroasiatic people (McColl et al., 2018). Paleolithic genomes from Tianyuan Cave and Amur River Basin also revealed a complex genetic admixture landscape in northern East Asians since 40 kya (Mao et al., 2021). Yang et al. (2020) recently analyzed the genetic structure and population shift or admixture history of ancient northern and southern East Asians dating back to 9,500–300 years ago. They found that the gene flow among these populations had made contributions to the genetic patterns of all present-day East Asians since the Neolithic (Yang et al., 2020). Wang C. C. et al. (2021) also genotyped and analyzed the most comprehensive set of ancient genomic data from northern, central, and southern East Asia and reconstructed four Holocene population expansion events that shaped the modern genetic diversity of eastern Eurasians, including three eastern migration events spread Languages of Sino-Tibetan, southern Chinese multi-families and Altaic to the surrounding areas with the Yellow River Basin, Yangtze River Basin, and Mongolian Plateau as the centers and one western Eurasian eastward dispersal, which was consistent with the genetically attested population movements accompanied by subsistence shifts (Ning et al., 2020). However, the extent to which ethnically/geographically diverse modern populations obtained ancestry from these ancient ancestral sources remained to be further characterized.

Chronologically and historically, the Han Chinese could trace their origins back to the Huang Di’s Tribe (Huaxia Tribe) in the central valley of the Yellow River about 5,000 years ago (China, 1995). Based on the millenarian antiquity of war and politics, the long-range evolution of agriculture technology, and the admixture movement of south–northern population migration, the Han Chinese gradually developed and formed *via* the indigenous populations with northward or southward incomers (China, 1995; Zhao et al., 2015). Subsequently, the northern Han Chinese embarked on a long-range period of continuous southward diffusion across various channels due to other political wars and natural famine over the past two millennia (Chen et al., 2009). They concurrently posed massive genetic admixtures with the native dwellers. Thus, the Han Chinese, together with surrounding indigenous residents and their ancestors, have played a predominant role in shaping the genetic diversity of East Asians. Previous analyses of the North-to-South Han population structure were systematically explored by both uniparental markers (Y-chromosomal polymorphisms and mitochondrial DNA variations) (Wen et al., 2004; Wei, 2011; Chiang et al., 2018) and genome-wide autosomal SNPs with limited samples or direct co-analysis with the available ancient East Asians (Chen et al., 2009; Cao et al., 2020), which could only demonstrate a close correlation between geographical distribution and genetic structure categories or explore the extent of the impact of the demic or cultural diffusion on the formation of modern East Asians (Wen et al., 2004). Currently, the available ancient DNA studies found the genetic continuity among spatiotemporally diverse people in East Asians (Yang et al., 2020; Mao et al., 2021; Wang C. C. et al., 2021); thus, comprehensively representative modern publicly available datasets and available statistical methods [shared alleles in *f*-statistics (Patterson et al., 2012), shared haplotypes in fineSTRUCTURE v4 (Lawson et al., 2012) and GLOBETROTTER (Hellenthal et al., 2014), and shared haplogroup lineages] provided more information and the possibility to characterize a more complete picture of population origin, isolation, migration, and admixture processes of Han Chinese.

Guizhou, located in the southwestern region of China, has been documented to be an indispensable place with substantial sociocultural, genetic, and linguistic diversity, and forms the characteristics of a mountainous province with the densest population distribution. Guizhou is an important part of the Yungui Plateau, which is geographically close to ethnolinguistically diverse provinces of Yunnan, Guangxi, and Hunan in the southwest, south, and east, and to Chongqing and Sichuan in the northwest and north. The population in Guizhou is nowadays widely distributed among 18 local minorities including Miao, Bouyei, Dong, Tujia, Yi, Hui, Bai, Yao, Zhuang, Mongolian, Mulam, and Qiang. Han Chinese demographically accounted for the largest population in Guizhou ethnic groups and occupied more than 60% of the Guizhou population officially recognized by the local government (Chen et al., 2018). The overall language landscape of the Chinese population is dominated by more than 10 mainly language families (including Tai-Kadai, Hmong-Mien, Tungusic, Indo-European, Austroasiatic, Austronesian, Turkic, Mongolic, Koranic, and Sino-Tibetan) (Cavalli-Sforza, 1998). However, Guizhou populations are reported to be dominated by the Tai-Kadai, Hmong-Mien, and Sino-Tibetan-speaking families (Chen et al., 2018; Liu et al., 2020). A large number of population genetic researches have been conducted and primarily focused on the forensic polymorphism and genetic structure of the Guizhou Hans *via* STRs included in the AGCU X19 amplification system or InDels included the Investigator DIPplex kit (Chen et al., 2018; He et al., 2019; Liu et al., 2020). The previous genetic analyses focusing on Guizhou populations suggested that the genetic variations of Guizhou Hans were associated with geographical divisions and linguistic classifications. However, genome-wide SNP data have not been provided to investigate the fine-scale genetic structure of southwestern Hans in this ethnolinguistic region. Besides, we also found that ancient DNA in southwestern China is lacking due to the humid and acid–base environment which is not conducive to the preservation of ancient DNA (Yang et al., 2020; Mao et al., 2021; Wang C. C. et al., 2021). Thus, more genome-wide data of geographically denser modern populations and comprehensive population genetic analysis with the surrounding ancient genomes could provide some new insights into historical and prehistoric demographic processes of Guizhou populations. To this end, we generated and analyzed genome-wide SNP data of more than 700,0000 genome-wide SNPs from 50 Han samples across four regions in Guizhou Province to explore the population structure and genetic admixture of the Guizhou Hans and to identify candidate loci targeted for positive natural selection.

## Results

### General Patterns of the Population Structure

**FIGURE 1:**
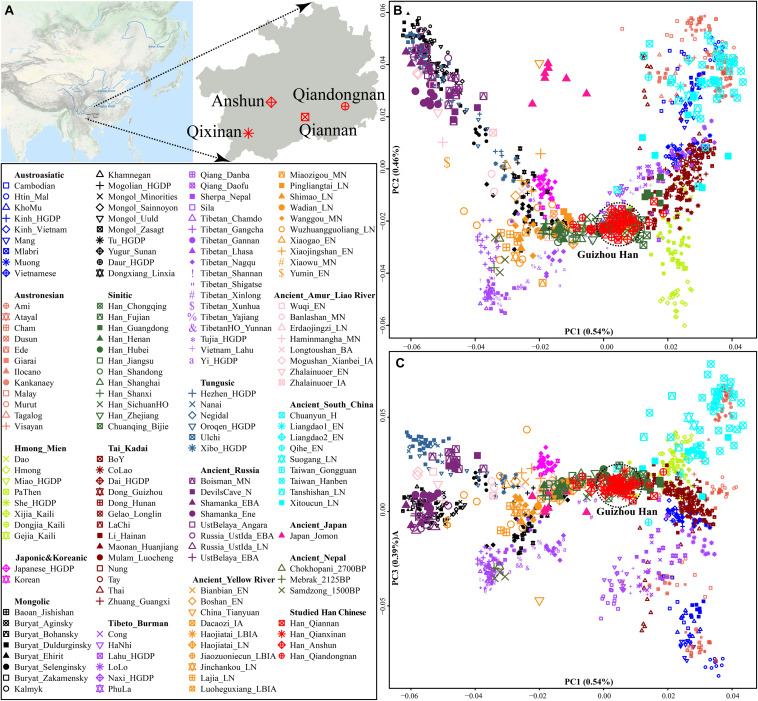
General information of Guizhou Hans. **(A)** The geographic positions of four studied Guizhou Hans in southwestern China. **(B,C)** Principal component analysis based on the top three components among 1,220 modern and ancient East Asians from Altaic, Sino-Tibetan, Austronesian, Austroasiatic, Hmong-Mien, and Tai-Kadai people and other ancient eastern Eurasians (which were projected onto the basic background). Populations were shaped according to their unique ID from one language family. Different populations from one language families or groups were colored.

We collected and generated genome-wide data of 50 Han Chinese individuals from Guizhou Province (Figure 1A and Table 1) and merged it with population data of over 3,000 modern Eurasians (mainly including Altaic, Sino-Tibetan, Austronesian, Austroasiatic, Hmong-Mien, and Tai-Kadai speakers within and around China) and ancient Eastern Eurasians from Nepal, China, Mongolia, Russia, Japan, and others^1^ (Jeong et al., 2016; Ning et al., 2020; Yang et al., 2020; Chen et al., 2021; Liu et al., 2021b; Wang C. C. et al., 2021; Yao et al., 2021). We conducted a principal component analysis (PCA) based on the modern population dataset and projected ancient populations onto the basic framework constructed based on the modern genetic variations. We found that ancient populations from Mongolia and Russia formed a cline with modern Tungusic and Mongolic-speaking populations (modern/ancient northeastern Asian cline). Four studied Guizhou Hans were localized between the Tibeto-Burman-speaking population cluster and one meta-population cluster from southern China consisting of Austronesian, Austroasiatic, Tai-Kadai, and Hmong-Mien-speaking people (Figure 1B). Four Han Chinese populations were clustered tightly and deviated to southern East Asians compared with northern Han Chinese populations from Shaanxi, Shanxi, and Shandong provinces. Furthermore, Guizhou Hans had a close genetic relationship with other reference Han people and neighboring Tai-Kadai populations compared with the geographically close Guizhou Hmong-Mien-speaking Gejia, Dongjia, and Xijia people. Compared with the ancestry composition of geographically close Chuanqing people (Lu et al., 2020), four Han Chinese from Anshun, Qiannan, Qianxinan, and Qiandongnan cities harbored more shared ancestry related to northern Han Chinese populations. Patterns of the genetic relationship inferred from the first and third components showed the separation between Austronesian and Austroasiatic people (Figure 1C). Guizhou Hans still overlapped with southern Han Chinese populations and had a close genetic relationship with Hmong-Mien and Tai-Kadai people on this scale.

**TABLE 1:** The demographical information and paternal and maternal haplogroups of our included 50 Han Chinese individuals.

Ind	Group	Status	MtDNA Haplogroup	Sex	Y-chromosome Haplogroup	Key Y-mutations
N0726	Han_Qiannan	Unrelated Healthy	Z3	Female	NA	NA
N0738	Han_Anshun	Unrelated Healthy	B5a1c1	Female	NA	NA
N0741	Han_Anshun	Unrelated Healthy	B5a1c1	Female	NA	NA
N0743	Han_Anshun	Unrelated Healthy	D5b1c	Male	O2a1c1a1a1a1e1a	[′18404653 Y16154′]
N0745	Han_Anshun	Unrelated Healthy	N9a4	Male	O2a1c1a1a1a1a1a1b	①
N0748	Han_Anshun	Unrelated Healthy	B5a1c1	Male	O2a2a1a2a1a	[′16880955 F2309,′ ′19566267 F3085′]
N0749	Han_Anshun	Unrelated Healthy	F1a′c′f	Male	O1b1a1a1b1a	⑤
N0750	Han_Anshun	Unrelated Healthy	B4	Female	NA	NA
N0751	Han_Anshun	Unrelated Healthy	B5a1c1	Female	NA	NA
N0753	Han_Anshun	Unrelated Healthy	D4a5	Female	NA	NA
N0754	Han_Anshun	Unrelated Healthy	C4a1′5	Female	NA	NA
N0764	Han_Anshun	Unrelated Healthy	F1a1	Female	NA	NA
N0765	Han_Anshun	Unrelated Healthy	M7b1a1	Female	NA	NA
N0775	Han_Anshun	Unrelated Healthy	B5a1c1	Female	NA	NA
N0733	Han_Anshun	Unrelated Healthy	D5a3	Female	NA	NA
N0735	Han_Anshun	Unrelated Healthy	D4j11	Male	O2a1c1a1a1a1e2a	[′15953241 FGC54507′]
N0736	Han_Qiandongnan	Unrelated Healthy	B4a2b	Female	NA	NA
N0740	Han_Qiandongnan	Unrelated Healthy	F1a1d	Female	NA	NA
N0742	Han_Qiandongnan	Unrelated Healthy	M7a1a	Male	O1b1a1a1a1a1a1b	[′2738084 Z24091,′ ′23959373 Z24093′]
N0746	Han_Qiandongnan	Unrelated Healthy	D4b2b	Female	NA	NA
N0747	Han_Qiandongnan	Unrelated Healthy	F1a1	Female	NA	NA
N0752	Han_Qiandongnan	Unrelated Healthy	F4a2	Female	NA	NA
N0761	Han_Qiandongnan	Unrelated Healthy	B5a1c1	Female	NA	NA
N0770	Han_Qiandongnan	Unrelated Healthy	B4h1	Male	O2a1c1a1a1a1a1a1a1a1	[′22548606 F1495,′ ′23976986 F1418′]
N0771	Han_Qiandongnan	Unrelated Healthy	D4a3b2	Female	NA	NA
N0774	Han_Qiandongnan	Unrelated Healthy	D4	Female	NA	NA
N0727	Han_Qiannan	Unrelated Healthy	B4a2b	Male	O2a2b1a2a1a2	①
N0737	Han_Qiannan	Unrelated Healthy	D4e1a2	Male	O1b1a1b1	③
N0739	Han_Qiannan	Unrelated Healthy	D5a2a1	Female	NA	NA
N0744	Han_Qiannan	Unrelated Healthy	M7b1a1	Female	NA	NA
N0728	Han_Qiannan	Unrelated Healthy	M7c3	Male	O2a1c1a1a1a1a1a1b	②
N0755	Han_Qiannan	Unrelated Healthy	M9b	Male	O2b1a	④
N0729	Han_Qiannan	Unrelated Healthy	M9b	Female	NA	NA
N0756	Han_Qiannan	Unrelated Healthy	F1a1	Female	NA	NA
N0757	Han_Qiannan	Unrelated Healthy	C7	Male	O1a1a1b2a1	[′8598326 Z39268,′ ′22908919 SK1571′]
N0758	Han_Qiannan	Unrelated Healthy	B5b2c	Male	O2a2b1a2a1a2	①
N0772	Han_Qiannan	Unrelated Healthy	B5a1c1	Female	NA	NA
N0773	Han_Qiannan	Unrelated Healthy	F1a1	Male	O2a1a1a	[′14928001 F1867′]
N0731	Han_Qiannan	Unrelated Healthy	B4c1a	Male	O2a2b1a2a1a2	①
N0734	Han_Qiannan	Unrelated Healthy	G2a	Male	O2a2b1a1a	[′2800495 F8,′ ′6840710 F42′]
N0759	Han_Qianxinan	Unrelated Healthy	B5a	Male	O2a1c1a1a1a1a1a1b	②
N0760	Han_Qianxinan	Unrelated Healthy	B4a	Female	NA	NA
N0762	Han_Qianxinan	Unrelated Healthy	F1c1a1	Female	NA	NA
N0763	Han_Qianxinan	Unrelated Healthy	B5a1c1	Female	NA	NA
N0730	Han_Qianxinan	Unrelated Healthy	B5a1	Female	NA	NA
N0766	Han_Qianxinan	Unrelated Healthy	F1d1	Female	NA	NA
N0767	Han_Qianxinan	Unrelated Healthy	F1a2a	Female	NA	NA
N0768	Han_Qianxinan	Unrelated Healthy	F1a2a	Female	NA	NA
N0769	Han_Qianxinan	Unrelated Healthy	B4b1a2	Male	D1a1a1a1a2a∼	[′16411247 Z44637′]
N0732	Han_Qianxinan	Unrelated Healthy	M10a1b	Male	O1a1a1a1a1a1b1	[′23159740 CTS11553′]

*① [′14173991 F242,′ ′14946079 F273,′ ′19371700 CTS10286,′ ′19436515 CTS10401,′ ′22821767 CTS10888,′ ′23024318 F634′].*

*② [′6655747 F793,′ ′7316384 Z43869,′ ′8568806 F1316C,′ ′14746939 Z43872,′ ′15912372 F2035,′ ′16253175 F2108,′ ′18241027 Z43875,′ ′18705724 Z43876,′ ′21283525 Z43877′].*

*③ [′8562287 F1309,′ ′14065037 F1685,′ ′14263051 F1742,′ ′16521659 F2189,′ ′17326573 F2445,′ ′22742290 F3353′].*

*④ [′14336908 F1770,′ ′16718236 F2244,′ ′16733921 F2247,′ ′22676898 F3338′].*

*⑤ [′15097700 B426,′ ′15320317 Z23672,′ ′16794293 Y9320,′ ′18816557 FGC29898′].*

**FIGURE 2:**
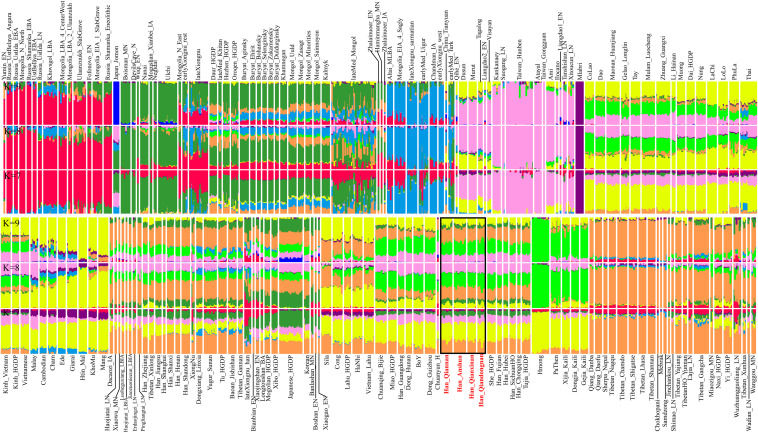
Model-based analysis result among 1,471 individuals in 179 eastern Eurasian populations. The visualized plots used the ancestry coefficient when seven to nine ancestral sources were predefined.

Furthermore, we further explored the ancestry composition based on the model-based ADMIXTURE analysis (Figure 2), which fitted the gene pool of the targeted populations using the unlinked SNP data with specific predefined ancestral sources (2–20). Here, we observed the relatively low cross-validation errors when the *K*-values were equal to 7∼9 (Supplementary Figure 1). We could observe four major ancestries in Guizhou Hans when seven ancestral sources were used: pink ancestry maximized in Taiwan Neolithic to Iron Age populations (Hanben and Gongguan) and modern Taiwan indigenous Austronesian Ami people; yellow ancestry dominant in Hmong-Mien-speaking Hmong and PaThen and some Austroasiatic populations; orange ancestry existed in modern Tibeto-Burman-speaking Tibetan and Qiang with high proportion; and limited green ancestry maximized in coastal Amur River Neolithic populations related to Boisman, DevilsGate, and modern Ulchi. Guizhou Hans harbored more Hmong-related ancestry compared with northern Hans (Henan, Shanxi, and Shandong Hans), and geographically close Neolithic to historical ancients in Henan possessed more Tibetan/Qiang-related ancestry. However, ancient people from Haojiatai, Xiaowu, and Pingliangtai had less Hmong-related ancestry compared with the geographically close modern northern Hans, which suggested that population movements and admixture shaped the spatiotemporal landscape in this region. We also found more Tibetan/Qiang ancestry in Guizhou Hans compared with their neighboring indigenes (e.g., Dong). When we used the increased predefined ancestral sources in the ancestry composition modeling, southern inland East Asian ancestries associated with Hmong-Mien and Tai-Kadai people were separated, and both contributed to the ancestry compositions of four Guizhou Hans, which suggested that our four studied Han Chinese populations were mixture populations and obtained gene flow from multiple southern indigenous ancestral populations.

### Genetic Origin and Admixture History Inferred From the Shared Ancestry in Allele-Based Analysis

To explore the intra/interpopulation genetic relationships between four genotyped Hans and other references (both modern and ancient populations), we calculated pairwise Fst genetic distances among 87 East Asian groups (Supplementary Table 1). We did not identify a genetic difference within the four studied populations, as we observed the most negative or smaller index values within four Hans. Compared with other adjoin populations, Qiannan Hans had a close genetic relationship with central and southern Hans (Hubei Hans: 0.0002, Sichuan Hans: 0.0003, Fujian and Zhejiang Hans: 0.0013). The other three Han Chinese populations possessed similar patterns of genetic correlation with our included reference populations, as the observed high correlation coefficient (larger than 0.99). We also assessed genetic affinity *via* the shared genetic drift measured from the outgroup *f*_3_-statistics in the form *f*_3_(reference modern and ancient populations, targeted populations; Mbuti). We first confirmed the close genetic relationship between Guizhou Hans and southern Han Chinese as the identified largest *f*_3_-values among them (Supplementary Table 2), and then we observed affinity between Guizhou Hans and geographically close Hmong-Mien-speaking populations (0.3036 between Anshun Hans and Kali Gejias, 0.3047 between Kaili Gejias and Qiandongnan Hans, 0.3029 between Kaili Gejias and Qiannan Hans, and 0.3030 between Kaili Gejias and Qianxinan Hans). Among ancient reference populations, we also identified the largest values between studied Hans and Taiwan Gongguan and Yellow River Luoheguxiang ancient populations.

We further performed admixture *f*_3_-statistics in the form *f*_3_(source1, source2; targeted populations) to identify and describe plausible admixture events. We identified 4,451 pairs that showed statistically negative *f*_3_-values in Anshun Hans which denoted that the allele frequency of the targeted Anshun Hans intermediated between that in source1 and source2. The observed significant *f*_3_-values suggested that Anshun Han was a genetic admixture population. The most negative source pairs were produced from northern Hans and southern East Asian Tai-Kadai people (Supplementary Table 3). The plausible proxies of southern ancestral sources were related to Austronesian and Hmong-Mien-speaking populations, as negative values in *f*_3_(northern sources, Austronesian/Hmong-Mien; Guizhou Hans). Northern Hans-linked ancestral sources were also related to Tibeto-Burman people and some of the Altaic-speaking populations. Indeed, these population pairs of one source from Tibeto-Burman or Altaic-speaking populations and the other from Austronesian and Tai-Kadai groups also produced significant admixture signals. Ancient people from Mongolia and the Yellow River Basin could also be used as the candidates of northern sources, and ancient populations from Fujian and Taiwan in southeastern coastal regions and Southeast Asia also could be used as effective southern ancestral sources for four Guizhou Hans. We conducted 30,288 pairs of symmetric *f*_4_-statistics in the form *f*_4_(Eurasian1, Eurasian2; targeted Guizhou Hans, Mbuti) and found more shared ancestry between Guizhou Hans and Tai-Kadai-speaking populations (Supplementary Table 4), such as the most negative tests of *f*_4_(Giarai, Zhuang_Guangxi; Han_Anshun, Mbuti) = –25.375^∗^SE. Compared with modern coastal southern East Asians related to Amis, Guizhou Hans shared more alleles with inland modern southern East Asians related to Hmong, *f*_4_(Amis, Hmong; Han_Qiandongnan, Mbuti) = –2.818, which was consistent with the observed patterns compared with Taiwan Hanben (*Z*-scores = –5.382). Affinity *f*_4_-statistics in the form *f*_4_(Eurasain1, Targeted Guizhou Hans; Eurasain2, Mbuti) were conducted to explore if some additional ancestries contributed to Guizhou Hans compared with other Eurasian comparative subjects. Compared with geographically close Guizhou Dong, Guizhou Hans harbored more ancestry related to Tibeto-Burman-speaking Tibetan (Chamdo, –5.274), as well as related to the Yellow River Basin ancient populations of middle Neolithic Wanggou people (–4.364), suggesting that more northern East Asian ancestry existed compared with southern indigenous people. Compared with Yellow River farmers (Haojiatai_LBIA), Guizhou Hans also possessed more ancestry related to southern East Asian indigenous populations related to Mlabri (–4.016) and others, which suggested that Guizhou Hans were formed with the gene pool from northern and southern sources.

**FIGURE 3:**
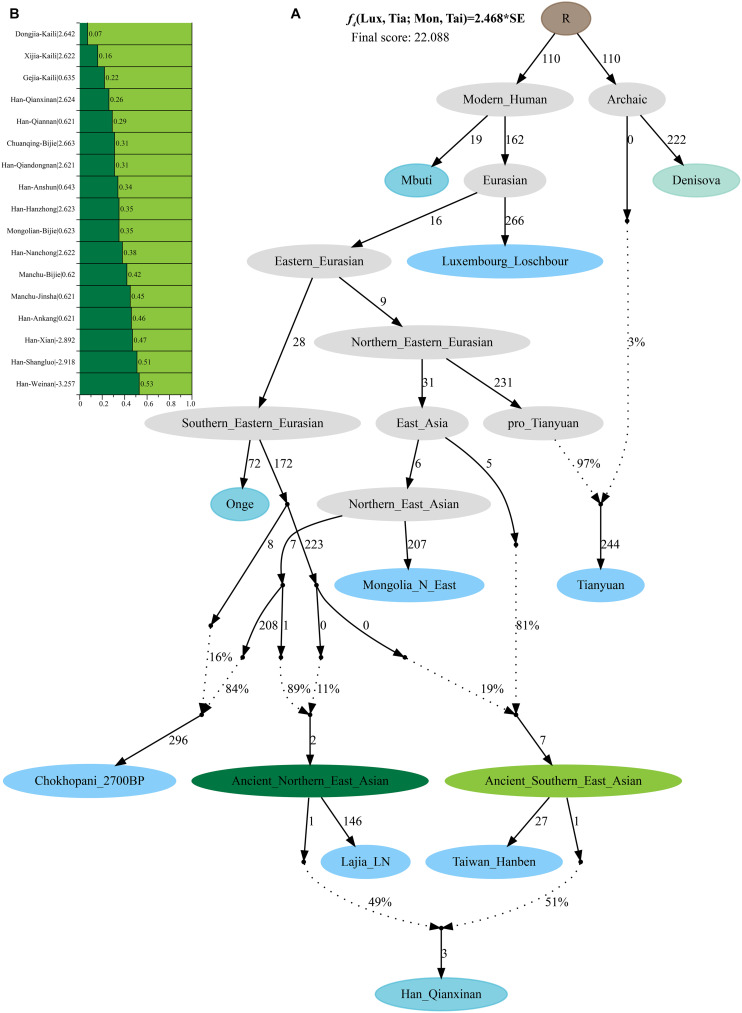
Deep population admixture history reconstruction with the population splits and admixture events. **(A)** The qpGraph-based phylogenetic framework focused on the formation of Qianxinan Hans. **(B)** Admixture proportion and corresponding lowest *Z*-scores of the qpGraph models for other Hans and Guizhou aborigines. Different features of populations (ghost, archaic, ancient, and modern populations) were color-coded. Admixture events were denoted with dotted lines and marked the corresponding admixture proportion on it. Branch length was measured using *f*_2_-values (1,000×).

**FIGURE 4:**
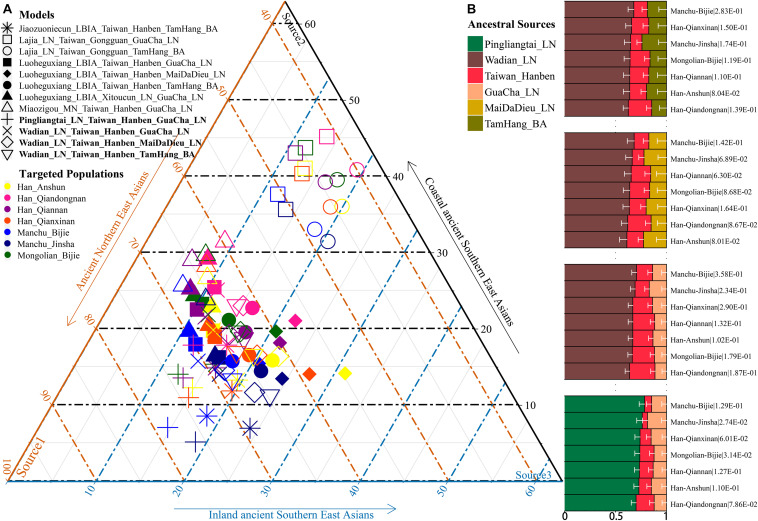
Three-way admixture models for seven Guizhou populations. **(A)** Variable ancestry proportion of three ancestral sources under the 12 fitted admixture models. **(B)** Histogram showed the major ancestry of Guizhou populations derived from the Yellow River farmers. White bar denoted the standard errors of the ancestry proportion. *p*-values of the tests were labeled behind the population name.

**FIGURE 5:**
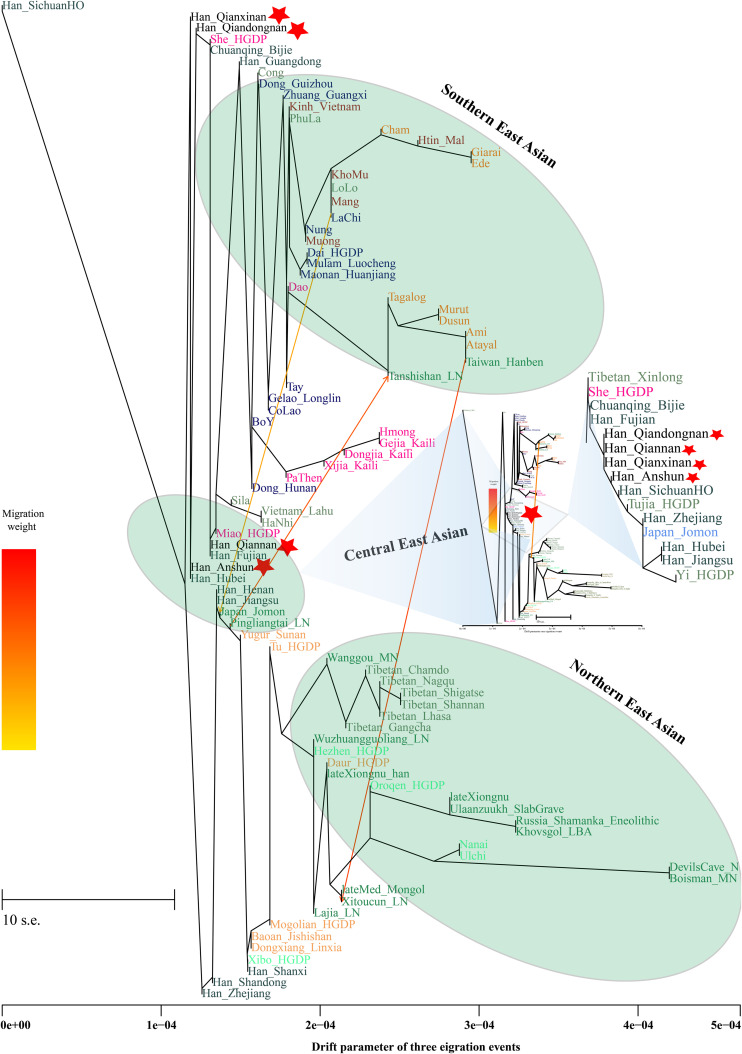
TreeMix-based phylogenetic relationship between four Guizhou Hans and other modern and ancient East Asians with three migration events. Different populations from one language families/groups were color-coded.

Additionally, we reconstructed deep population admixture models for the formation of Guizhou Hans *via* qpGraph-based phylogeny framework with population split and admixture events (Figure 3A). Here, we used the late Neolithic Qijia culture-related ancient population as the northern ancestral source and used Neolithic to Iron Age Hanben people from southeastern China as the southern ancestral source. All four studied populations could be successfully fitted in this model with fluctuated proportions, which showed that the southwestern Hans from Guizhou Province harbored both northern and southern ancient East Asian ancestries. Totally, admixture processes kept a similar pattern with Chuanqing people from Guizhou Bijie City (modeled as the admixture of 0.31 of their ancestry related to Lajia and the remaining ancestry from Hanben). Compared with northern Hans from Shaanxi Province and recent southward Manchus and Mongolians in Guizhou Province, ancestral proportion related to the northern Lajia decreased in Guizhou Hans, but increased compared with Guizhou indigenous Hmong-Mien speakers (Gejia, Dongjia, and Xijia, Figure 3B). To further explore whether a differentiated genetic contribution from coastal and inland southern East Asians, we followingly conducted three-way admixture models with two sources from southern East Asia (inland and coastal) and one from northern East Asia focused on the four studied Guizhou Hans and four published Guizhou minorities (Figure 4). The putative northern sources included middle and upper Yellow River farmers of the Jiaozuoniecun_LBIA, Lajia_LN, Luoheguxiang_LBIA, Miaozigou_MN, Pingliangtai_LN, and Wadian_LN; the coastal southern sources comprised Neolithic to Iron Age populations (Taiwan_Hanben, Taiwan_Gongguan, and Xitoucun_LN); and inland sources were made up of Neolithic to Bronze Age populations of TamHang_BA, GuaCha_LN, and MaiDaDieu_LN. We obtained 603 fitted admixture models with major ancestry from northern sources and the second from the southern coastal sources and the last from the inland southern sources (Supplementary Table 5). It should be also noted that the putative inland southern East Asian ancient sources possessed some extent ancestry related to the indigenous Hòabìnhian hunter-gatherers (McColl et al., 2018), which may be biased against the true estimated proportion of inland ancestral sources. Ancient DNA from Daxi, Shijiahe, and other southwestern ancient people in the future will provide better fitted and more explicable admixture models for Guizhou Hans. Here, we also constructed one neighboring-joining phylogenetic tree among 86 modern and ancient eastern Eurasians. The unrooted tree was divided into two branches: the northern one comprised Mongolic and Tungusic speakers and Neolithic to Iron Age populations from Yellow River Basin, Mongolia Plateau, and southern Siberia and the southern branch was made up of Austronesian, Austroasiatic, and Hmong-Mien populations from southern China and Southeast Asia (Figure 5). Guizhou Hans and other Han Chinese groups were clustered between these northern and southern branches and had a close phylogenetic relationship with each other and then clustered with geographically close Sichuan, Hubei, and Fujian Hans.

### Finer-Scale Population Substructure and Natural Selection Signatures Revealed *via* the Sharing Haplotype Based on the Linked Successive SNPs

**FIGURE 6:**
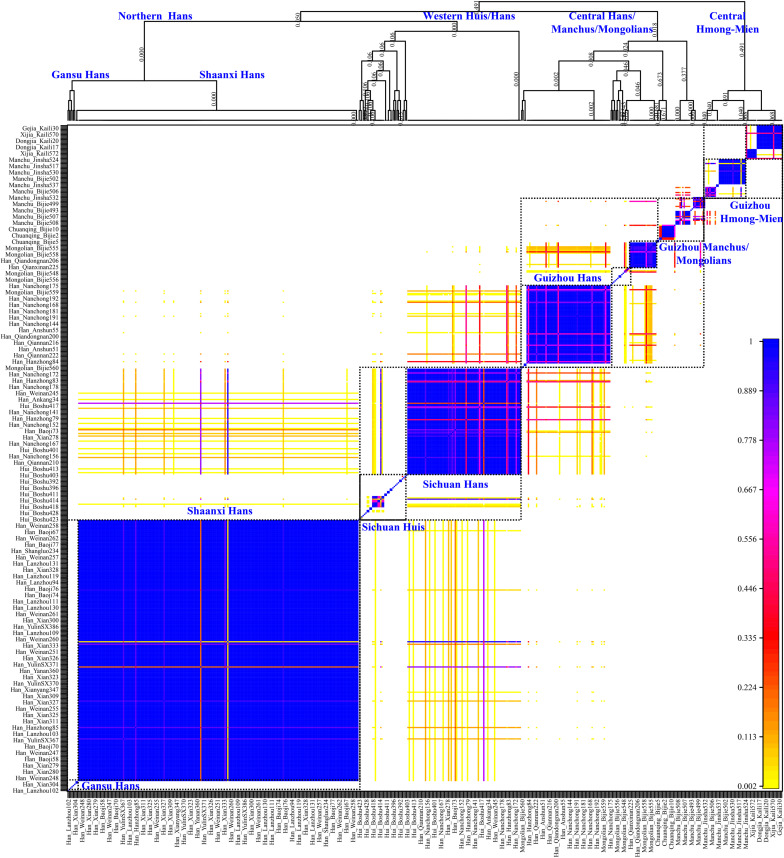
Genetic structure of Chinese populations from haplotype analysis using fineSTRUCTURE. The top parts showed the dendrogram of the genetically homogeneous populations and the bottom heatmap showed the pairwise coincidence. No meaning of the branch lengths but the hierarchy denoted the branching pattern clustered based on the haplotype sharing.

Genetic analysis based on the unlinked SNPs can only capture the major information of population history encoded in the genomes. Thus, to further identify, date, and describe the fine-scale admixture events and decode more detailed information of population demographic history of Guizhou Hans, we merged population data with previous published East Asians genotyped using the same array (700K), including 11 Han populations from Shaanxi Province (He G.-L. et al., 2020), Lanzhou Hans from Gansu Province (Yao et al., 2021), Boshu Huis and Nanchong Hans (Liu et al., 2021b) from Sichuan Province, officially unrecognized populations [Chuanqings, Gejias, Dongjias, and Xijias (Lu et al., 2020)], and Manchus and Mongolians from Guizhou Province (Chen et al., 2021). We used SHAPEIT to phase the genome-wide data and obtain the phased SNPs. We first used the chromosome painting strategy instrumented in the ChromoPainter to paint the chromosome of the targeted population conditional on all potential DNA donors and to choose the best ancestry source based on the co-ancestry matrix. Both individual-level shared length and number of haplotype chunks were obtained. Moreover, it showed that the Guizhou Hans shared large and long ancestry fragments (also referred to as identity by the decedent, IBD) with geographically close populations, suggesting that Guizhou Hans had the most common ancestor among them. Furthermore, we used ChromoCombine to combine the shared number of ancestry fragments and converted the differentiated contributed haplotype states from all possible donors to the focused targeted populations as the co-ancestry matrix. Fine-scale population structure was characterized from individuals within populations using fineSTRUCTURE and grouped them into new genetically defined groups based on the genetic similarity. Here, the heatmaps of pairwise coincidence, co-ancestry, and average co-ancestry were visualized and used to explore the genetic background. Dendrograms and PCA clustering patterns based on the co-ancestry matrix were also conducted. We observed more subtle population substructures among northern Hans, western Hans, and Central Hans, as well as the clear population stratification between Guizhou Hans and Guizhou indigenous minorities (Gejia, Chuanqing, Dongjia, and Xijia), Huis, Mongolians, and Manchus (Figure 6). Most of the inferred genetically homogeneous populations were consistent with the geography-based defined population labels. All included individuals were classified into northern Shaanxi Hans, western Sichuan Hans, and southwestern Guizhou Hans and other minority clusters (Sichuan Huis, Guizhou Mongolians/Manchus, and Guizhou Hmong-Miens). Four Guizhou Hans formed into one branch and clustered with some Nanchong Hans, suggesting the homogenization of Guizhou Hans and the close genetic connection between southwestern Hans and western Hans (may be associated with the historically documented HuGuang Ruchuan migration).

Autosomal haplotype data also could provide new insights into the population admixture history. Previous genetic analyses focused on the shared haplotype have reconstructed the admixture sources and processes of Bantu expansion and Arab slave trade in Africa, the Mongol Empire and the first millennium CE migrations in Eurasia (Hellenthal et al., 2014). Here, we used the reconstructed shared haplotype chunk length from ChromoCombine to further explore the possible admixture events. We used genetically similar groups as the proxy of the true admixture source and employed northern Shaanxi Hans as the possible ancestral northern donors and Guizhou minorities as the southern source donors. We used GLOBETROTTER to analyze 573 individuals from 23 Chinese populations. Strong evidence of admixture was observed in four targeted populations (*p* < 0.05). Anshun Hans were inferred as the one-date admixture results in the best-guess inference, which was mixed from 0.17 haplotypes from local Kaili Xijia people and other 0.83 from northern Hanzhong Hans occurred around 13 generations ago. A similar pattern of one-date admixture model was also obtained in others with similar best-guess ancestral sources, admixture dates, and corresponding proportions, such as Qiannan Hans that were mixed with minor ancestry (0.2) from Xijia and major ancestry (0.8) from Hanzhong Hans at 15 generations ago.

**FIGURE 7:**
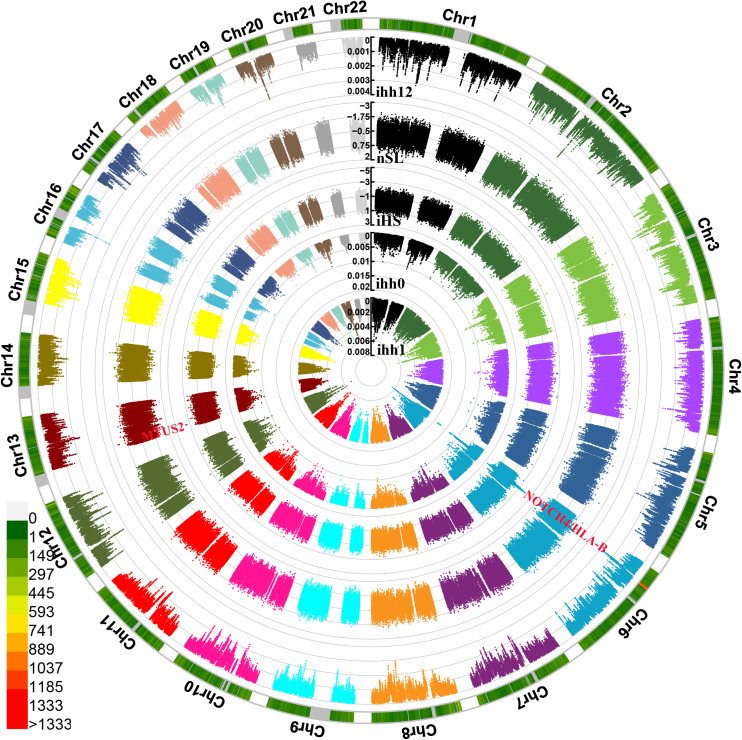
Circle Manhattan plots showed the signatures of natural selection based on the different statistic indexes. From inside to outsides: allele frequency of ancestry allele, ihh1, ihh0, iHS, nSL, and ihh12.

We also identified and characterized the plausible exiting natural selection signals in Guizhou Hans based on the phased haplotypes using the integrated haplotype score (iHS), nSL, and integrated haplotype homozygosity pooled (iHH12). We used 329,863 phased loci from 100 haplotypes in Guizhou Hans (Figure 7). The most significant inferred selection signals were observed from chromosomes 6 and 13. These genes were associated with the susceptibility of complex diseases, including neurogenic locus notch homolog protein4 (NOTCH4) located in 6p21.3, MICB (Human MHC class I chain-related B gene), Microtubule-associated tumor suppressor candidate 2 (MTUS2), and others. Similar patterns of the natural selection signatures were further confirmed *via* the observed signatures from other natural selection indexes (nSL, iHH12, and iHH). Three SNPs from MTUS2 genes located in chromosome 13 (243198, 243199, and 243196) also possessed negative nSL values (–2.5738, –2.5716, and –2.4624).

**FIGURE 8:**
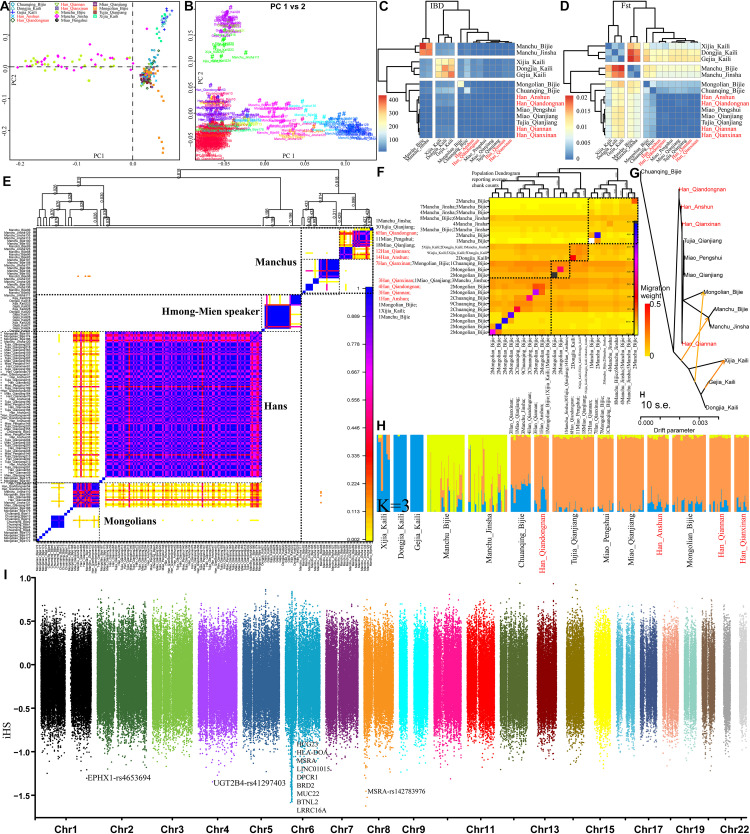
Finer-scale genetic characteristics within 28 southwestern populations. PCA results based on the allele frequency **(A)** and the shared haplotypes **(B)**. Heatmap was visualized based on the pairwise Fst genetic distance **(C)** and the shared IBD segments **(D)**; individual **(E)** or population **(F)** clustering patterns inferred from the co-ancestry matrix in the fineSTRUCTURE analysis; descriptive analysis results from the TreeMix-based phylogeny **(G)** and model-based admixture results **(H)**; natural selection signals inferred from the iHS **(I)**.

Finally, we focused on the genetic diversity and structure within 230 individuals from 14 Guizhou or Chongqing populations. PCA results based on the allele frequency distribution of single SNPs showed three genetic clines (Manchus, Mongolians, and Hmong-Mien speakers) and Guizhou Hans clustered in the intermediated position among three clines (Figure 8A), which was further confirmed with the patterns of genetic relationships in the haplotype-based PCA inferred from the co-ancestry matrix (Figure 8B). Pairwise IBD and Fst genetic distance visualized in the heatmap (Figures 8C,D) revealed the close genetic relationship between Guizhou Hans and geographically close Miao and Tujia and a distant relationship with Tungusic-speaking Manchus and Hmong-Mien-speaking Gejias, Dongjias, and Xijias. Finer-scale genetic structure inferred from fineSTRUCTURE based on the co-ancestry matrix among 14 populations showed that Manchus formed one genetically separated group and Guizhou Hans clustered closed with Mongolians, Tujias, and Hans than with Hmong-Mien-speakers in both individual- and population-level shared chunks (Figures 8E,F). These substructures among Chinese southwestern populations were also observed as the identified similar clustered patterns in the TreeMix-based phylogeny framework (Figure 8G) and model-based ADMIXTURE models (Figure 8H). Focused on this meta-population from southwestern China, natural selection signatures inferred from iHS showed different top signals, but most signals were observed in chromosome 6 (Figure 8I). The inferred loci here are also associated with the susceptibility of the disease.

### Admixture Signatures Inferred From the Shared Paternal and Maternal Lineages

We genotyped 3,746 maternal lineage informative SNPs (LISNPs) in 50 female Hans and 24,047 LISNPs in 19 male Hans (Table 1). We identified 36 different terminal maternal lineages with the frequency ranging from 0.02 to 0.16. B5a1c1 was the dominant maternal lineage in Guizhou Hans (8/50), followed by F1a1 (4/50), and M7b1a1, B4a2b, M9b, and F1a2a. These observed maternal lineages generally retained relatively high frequencies in southern East Asia and Mainland Southeast Asia. A total of 15 terminal paternal lineages were identified with the frequency ranging from 0.0526 to 0.1579. O2a1b1a1a1a1a1a1b1-F793/Z43869/F1316C/Z43872/F2035/F2108/Z43875/Z43876/Z4 3877 and O2a2b1a2a1a1a2-F242/F273/CTS10286/CTS10401/CT S10888/F634 were the dominant lineages in the studied Hans. The paternal lineage of O2a2a1a2a1a1-F2309/F3085 was also dominant in Guizhou Hans. We also identified one D lineage (D1a1a1a1a2a) and five O1b1a1. The Guizhou Hans with paternal lineages O2a1b1a1a1a1a1a1b1 and O2a2b1a2a1a1a2 might be the descendants of two Neolithic super-grandfathers: Oγ (O2a1b1a1a1a-F11) and Oβ (O2a2b1a2a1a-F46) (Yan et al., 2014). O2a2a1a2a1a1 is common in Hmong-Mien and Austroasiatic speakers (Xia et al., 2019). The infrequent D1a1a1a1a2a∼ is predominant in Tibetan-related populations (Shi et al., 2008; Qi et al., 2013), while O1b1a1 is mainly distributed in southern East Asian, Japanese, and Southeast Asian populations (Yan et al., 2011; Park et al., 2012; Kutanan et al., 2019; Lang et al., 2019).

## Discussion

### Genetic Origins, Migration, and Admixture History of Guizhou Hans

East Asia is considered to be a region enriched with tremendous cultural and genetic diversity. Researches focused on the peopling of East Asia from ancient genome perspectives showed different ancient genetic ancestries in southern and northern East Asia (Mao et al., 2021; Wang T. et al., 2021). Genetic findings based on the genome-wide SNP data of modern people also demonstrated that the north–south/east–west genetic substructure profiles and demic diffusion of Han Chinese populations shared the overall genetic diversity and demographical history of modern East Asians (Chen et al., 2009; Chiang et al., 2018). In detail, genome-wide association study results based on the Han Chinese populations from 26 administrative regions found substantially genetic differentiation among them, in which its intricate substructures corresponded roughly to the northern Hans, central Hans, and southern Hans. These genetically attested genetic patterns were consistent with historical immigration, cultural exchanges, and geographical characteristics (Xu et al., 2009; Yang et al., 2021). Besides, Yang et al. (2020) recently reported a genome-wide ancient genome study from northern and southern East Asia in the early Neolithic period and found that the population movement and genetic admixture involving northern East Asian ancestry spread southward into Southeast Asia during the Neolithic period, which transformed the genetic ancestry of southern China. Wang T. et al. (2021) sequenced the ancient genomes from Fujian and Guangxi and also identified three ancient ancestry components (Qihe, Longlin, and Hoabinhian) in South China and Southeast Asia, which contributed to the formation of local Early Neolithic people *via* migration and admixture but a limited contribution to modern Guangxi and surrounding populations. Although advanced in deep population history reconstruction of ancient East Asians, the direct genetic contribution from ancient populations to Han Chinese populations needed to be further characterized. A large number of studies have been conducted to explore the genetic structure of Han Chinese groups across China, which suggested that genetic homogenization existed among the Han populations and the genetic differentiation was identified among populations from different language families (Guan et al., 2020; Li et al., 2020; Luo et al., 2020; Wu et al., 2020). Although these signs of progress have been achieved, the genetic history of Guizhou Hans, genetic admixture, or population relationship with neighboring ethnicities was vastly underrepresented due to the lack of sampling of present-day people and comparison with prehistoric East Asian populations. Thus, we generated new genome-wide data over 700K SNPs in Guizhou Hans and merged it with publicly available genomic data from the early Neolithic to the modern populations, and we conducted one comprehensive study focused on the genetic structure and genetic history of the Guizhou Han populations.

Genetic origins and admixture history of four geographically different Guizhou Hans and the finer-scale substructure among Han Chinese based on the genome-wide data were the main studied focus. We also included minorities (Geijia, Dongjia, Xijia, Manchu, and Mongolian) residing in Guizhou Province and other Hans from the surrounding provinces as our references (Lu et al., 2020; Chen et al., 2021; Liu et al., 2021b). PCA and ADMIXTURE results clustered Guizhou Hans in the intermediated position between northern East Asians and southern East Asians, consistent with the clustering patterns in the TreeMix-based phylogeny and the geographical location. Guizhou Province is one of the ethnolinguistically diverse provinces; thus, non-Hans played an important role in the gene pool of the Guizhou populations (Chen et al., 2021). Thus, the effect of the genetic material of Guizhou indigene on the genetic composition of Guizhou Hans is interesting. Indeed, comparative results in the descriptive analysis and qualitative measures in the *f*-statistics, as well as the fineSTRUCTURE-based finer-scale admixture evidence and corresponding admixture proportion from the putative ancestral sources, consistently showed that Guizhou Hans were mixed populations and shared excess ancestry with northern Hans and Henan late Neolithic to Iron Age ancient populations associated with Longshan culture and their dependents and also showed significant genetic differentiation with geographically close southern indigenes. Our findings were consistent with archeologically attested population history of ancient southwestern China, which has suggested that the main components of the southern indigenous people in Guizhou Province may be the direct descendants of prehistoric people associated with southwestern ancients linked with Pengtoushan, Gaomiao, Daxi, Qujialing, Shijiahe, and other cultures (Yu and Li, 2021). Thus, we could identify more southern East Asian ancestry related to Hanben, Hmong, or Tai-Kadai people in Guizhou minorities compared with Guizhou Hans.

Another point of this work was the focus on the patterns of the fine-scale genetic structure of Han Chinese from northern (Shaanxi) and southwestern Hans (Guizhou) and minority ethnic groups (Guizhou) and natural selection signatures based on the reconstructed ancestral chunks. We identified genetic differences among Shaanxi, Sichuan, and Guizhou Hans, as well as the genetic distinction between Han Chinese and minorities *via* the fineSTRUCTURE-based population clustering patterns. We also identified the recent admixture time based on the ALDER and GLOBETROTTER. The inferred that differentiated admixture signatures may be the plausible explanation for the observed genetic differentiation between Guizhou Hans and their non-Han neighbors. In the qpGraph- and qpAdm-based admixture models, we identified all northern and southern Neolithic farmers who participated in the formation of Guizhou Hans, which was related to the descendants of Yangtze Valley farmers and Yellow River Basin farmers (Wang et al., 2020), suggesting population interactions between northern and southern China were more ancient than the simplified admixture models proposed by ALDER and GLOBETROTTER. Multiple and continuous waves of multiple sources may participate in the formation of ethnolinguistically diverse East Asians. Recent ancient DNA findings of differentiated allele sharing in affinity *f*_4_-statistics between modern East Asians and Neolithic farmers from the northern Yellow River Basin and southern Yangtze River Basin can provide some clues for these hypothesized complex admixture models (Yang et al., 2020), which should be further explored *via* more modern and ancient deep sequencing data and more complex and reasonable biostatistic methods. Additionally, the primary ancestry of Han Chinese in Guizhou Province from northern China was further evidenced *via* the *f*_4_-statistics and GLOBETROTTER-based admixture characterization, supporting much-shared gene ancestry between the southwestern Han Chinese and the present-day northern Sino-Tibetan populations (e.g., Sino-Tibetan and northern Sinitic-speakers), which provided more autosomal genetic supporting evidence for the common origins of Sino-Tibetan language and people from the Yellow River Basin in northern China (Zhang M. et al., 2019).

### Positive Natural Selection Based on Successive Linked SNPs

We had explored the genome-wide candidate loci targeted by natural selections based on the reconstructed haplotype data. Among the identified natural selection loci, the top hundreds of SNPs were located in NOTCH4 and HLA in chromosome 6, which was associated with the susceptibility of complex diseases. SNP rs9262558 linked to the HLA gene family located in chromosome 6 was recently evidenced by obvious positive natural selection in Taiwan Hans (| iHS| = 7.5), and it also showed similar signatures in Guizhou Hans (–3.1243) (Yan et al., 2014).

Interestingly, we did not observe the most significant selection signals in EDAR (ectodysplasin A receptor, which was associated with facial and hair morphology in East Asians) and SLC24A5 (pigmentation gene in western Eurasians). Among 11 functional SNPs located in EDAR in chromosome 2 (rs260674, rs12466509, rs3827760, rs10865026, rs260687, rs260690, rs260714, rs6542787, rs6750964, rs922452, and rs72939934 with iHS values from –04017 to 1.2616), the first three SNPs were likely subjected to natural selection in Guizhou Hans with iHS values larger than 1. All of them except SNP rs72939934 harbored nSL values ranging from 1.1930 to 1.4646. SNP rs3827760, one variation being recently evidenced harboring greater genetic differentiation between Gansu Huis and Hans (Ma et al., 2021), also had relatively high natural selection signals in Guizhou Hans (iHS: 1.1139 and nSL: 1.2567). Among 3,133 SNPs located in the Solute Carrier Family (SLC) genes, we identified 70 loci that had large iHS values larger than 1 and 527 SNPs less than –1. The most natural selection marker was SNP rs11966200 located in SLC44A4 in chromosome 6, which was associated with susceptibility of postlingual non-syndromic mid-frequency hearing loss (Ma et al., 2017). SNP rs4148211 located in ABCG8 (iHS: –0.1227 and nSL: 0.0538 in Guizhou Hans) was also one identified SNP that possessed high genetic differentiation between Huis and Hans (Ma et al., 2021), but which is not significant in our studied Hans. Among 83 SNPs, we identified 15 SNPs with the natural selection signals (iHS < –1) located in ABCG1 or ABCG2 associated with the regulation lipid metabolism, especially for SNP rs3788008 (iHS: –2.1567). Endothelial Per-Arnt-Sim (PAS) domain protein 1 (EPAS1) and Hypoxia-inducible factor prolyl hydroxylase (EGLN) were evidenced as the key mutations of human adaptation to the high-altitude environment (Yi et al., 2010). Here, we analyzed 58 SNPs located in these two genes in lowland Hans and we only identified that two SNPs (rs3733829 and rs10151526) in EGLN and four SNPs (rs4953361, rs59901247, rs7577700, and rs187821419) in EPAS displayed natural selection signals. Seven out of 22 candidate loci located in the ADH in chromosome 4 also showed high | iHS| scores, especially for four loci (rs1042026, rs2066701, rs2075633, and rs1229984) located in ADH1B, which was strongly associated with alcohol metabolism. Next, population genomic analysis with denser sapling with larger sample size based on the genotyping array, second-generation sequencing, and third-generation sequencing (nanopore sequencing) should be conducted to confirm our findings and explore more comprehensive admixture profiles of Guizhou Hans.

## Conclusion

We conducted one comprehensive population genomic analysis based on three types of shared ancestries (sharing alleles, haplotype, and uniparental lineage) among Guizhou Hans and all publicly available genome-wide data from the early Neolithic to the modern Eurasian populations. We explored and reconstructed the genetic origin, migration, and admixture history of Guizhou Hans, as well as illuminated the candidate loci targeted for positive natural selection. Our survey illuminated that the present-day Guizhou Hans mainly derived the major ancestry from the Yellow River millet farmers and also obtained additional admixture ancestry from an indigenous southern source related to Yangtze River rice agriculturalists. Genetic clustering analysis based on the sharing of IBD patterns and paternal and maternal lineages also demonstrated that the Guizhou Hans were located in the middle position of the North–South genetic gradient consistent with their geographical origin. Additionally, we identified great genetic differentiation between Guizhou Hans and northern Shaanxi Hans, as well as between Guizhou Hans and Guizhou indigenous non-Han people based on the fineSTRUCTURE-based shared ancestry fragments, although we found a strong genetic homogeneity within four Guizhou Han populations. Finally, we searched for signatures of positive selection in the Guizhou Hans by scanning for SNPs that displayed unusually long haplotype lengths using iHS and identified hundreds of loci located in chromosome 6 (including HLA and NOTCH4) associated with the susceptibility of the complex diseases and other loci located in EDAR, ADH1B, and ABCG2 associated with morphology formation, alcohol, and lipid metabolism.

## Materials and Methods

### Sample Collection and SNP Genotyping

Following the recommendations of the Helsinki Declaration of 2013 (Wilson, 2013), this work was approved by the Ethics Committee of Xiamen University (XDYX201909). We collected 50 saliva samples from 50 unrelated individuals from four cities in Guizhou Province (Anshun, Qiannan, Qianxinan, and Qiandongnan, Figure 1A). All participants in this study were needed to be indigenous Han people residing in Guizhou Province for at least three generations. A high-density SNP genotyping array of Infinium^®^ Global Screening Array (GSA) was used to genotype around 700K SNPs from both autosomal and uniparental chromosomes. Genotyping success rate and missing rate per loci or individual were further controlled *via* PLINK 1.9 following our recent similar work (Chang et al., 2015; Liu et al., 2021b). We merged our data with publicly available modern and ancient reference populations from the Human Origin dataset and the 1240K dataset^2^ as the low-density dataset used for allele-based analysis. We also merged the newly generated data with other Chinese populations genotyped using the same genotyping chip as the high-density dataset for the haplotype-based analysis, including Hans, Huis, Mongolians, Manchus, Gejias, Xijias, and Dongjias (Chen et al., 2021; Liu et al., 2021b; Yao et al., 2021).

### Allele-Based Population Genetic Analysis

Principal component analysis (PCA) among eastern Eurasian or other local-scale populations was conducted using the smartpca package (Patterson et al., 2012). Ancient populations from Mongolia, China, and other neighboring countries were projected onto the top two components. PLINK 1.9 (Chang et al., 2015) was used to calculate the pairwise Fst genetic distances. Model-based clustering analyses were conducted based on the PLINK-pruned unlinked data (–indep-pairwise 200 25 0.4) and conducted using ADMIXTURE 1.3.0 (Alexander et al., 2009) with the predefined ancestry sources ranging from 2 to 20. We run 100 times for each predefined admixture model. A shared genetic drift between Guizhou Hans and other reference populations was measured using the *qp3pop* packages (Patterson et al., 2012) with the tested form of *f*_3_(Guizhou Hans, reference populations; Mbuti); here, the central African Mbuti population was used as the outgroup. Allele-based admixture signatures were explored using the admixture statistics in the form *f*_3_(source1, source2; Guizhou Hans), in which the observed negative values with *Z*-scores less than –3 denoted a strong admixture evidence. Four population-based analyses (both affinity *f*_4_- and asymmetric *f*_4_-statistics) were conducted using the *qpDstat* package (Patterson et al., 2012) with the *f*_4_ model used. The number of ancestral sources and corresponding admixture proportions was calculated using *qpWave/qpAdm* packages (Patterson et al., 2012), and qpGraph-based phylogeny with admixture events was constructed using *qpGraph* (Patterson et al., 2012). We also built the phylogenetic tree based on the allele frequency using TreeMix (Pickrell and Pritchard, 2012). ALDER (Loh et al., 2013) was used to estimate the admixture times with the predefined ancestral sources.

### Haplotype-Based Population Genomic Analyses and Uniparental Haplogroup Assignment

We first phased all populations included in the high-density dataset using ShapeIT v2 (Browning and Browning, 2013). Four Guizhou Han Chinese populations were regarded as the targeted populations, and other reference populations were used as the surrogated populations. All targeted and surrogated populations were used as the donor populations to paint the used recipient populations using the chromosome painting strategies in the ChromoPainter v2 and ChromoCombine v2 (Lawson et al., 2012). FineSTRUCTURE v4 (Lawson et al., 2012) was used to reconstruct individual-based trees and group genetically homogeneous clusters based on the IBD number. We also used the GLOBETROTTER (Hellenthal et al., 2014) to explore, date, and characterize the admixture events based on the IBD length. We assigned and determined the terminal Y-chromosomal haplogroups using our in-house script based on the Y-chromosome tree resource version 2017 in the International Society of Genetic Genealogy (ISOGG)^3^. Maternal haplogroups were determined using HaploGrep 2 (Weissensteiner et al., 2016).

## Data Availability Statement

The original data contributions presented in the study are included in the article/Supplementary Material, further inquiries can be directly obtained from the corresponding author/s.

## Ethics Statement

This work was approved by the Ethics Committee of Xiamen University. The sample was collected with informed consent. The patients/participants provided their written informed consent to participate in this study.

## Author Contributions

C-CW, BZ, CL, and GH designed this study. MW, DY, and GH wrote the manuscript. MW, GH, XZ, ZW, H-YY, JL, and L-HW conducted the experiment. MW, GH, XZ, ZW, H-YY, JL, and L-HW analyzed the results. C-CW, BZ, CL, and GH revised the manuscript. All the authors reviewed the manuscript.

## Conflict of Interest

The authors declare that the research was conducted in the absence of any commercial or financial relationships that could be construed as a potential conflict of interest. The handling editor declared a past co-authorship with the authors GH, H-YY, L-HW, C-CW, BZ, and CL.

## Publisher’s Note

All claims expressed in this article are solely those of the authors and do not necessarily represent those of their affiliated organizations, or those of the publisher, the editors and the reviewers. Any product that may be evaluated in this article, or claim that may be made by its manufacturer, is not guaranteed or endorsed by the publisher.

## Footnotes

1
https://reich.hms.harvard.edu/allen-ancient-dna-resource-aadr-downloadable-genotypes-present-day-and-ancient-dna-data

2
https://reich.hms.harvard.edu/downloadable-genotypes-present-day-and-ancient-dna-data-compiled-published-papers

3
https://isogg.org/tree/index.html

## Supplementary Material

Supplementary Figure 1 The distribution of cross-validation errors.
