# Supplementary figures and images for: Fine-Scale Genetic Structure and Natural Selection Signatures of Southwestern Hans Inferred From Patterns of Genome-Wide Allele, Haplotype, and Haplogroup Lineages
Source: Front Genet. 2021 Aug 24;12:727821. doi: 10.3389/fgene.2021.727821 (PMC8421688; doi:10.3389/fgene.2021.727821)

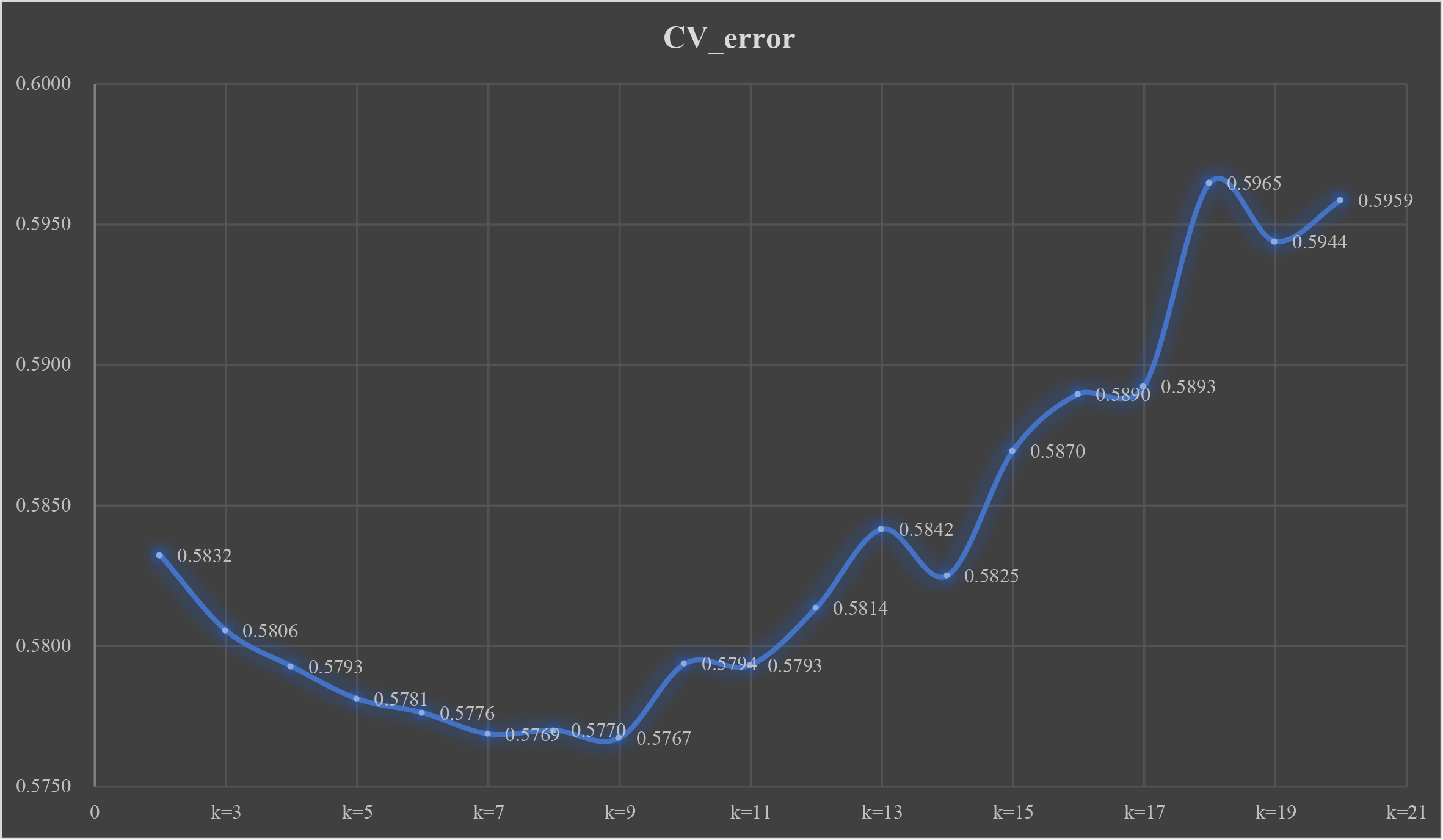

Supplement: Supplementary Figure 1 — The distribution of cross-validation errors. [file Image_1.TIF]
